# Task‐dependent responses to muscle vibration during reaching

**DOI:** 10.1111/ejn.14292

**Published:** 2018-12-11

**Authors:** Johannes Keyser, Rob E. F. S. Ramakers, W. Pieter Medendorp, Luc P. J. Selen

**Affiliations:** ^1^ Donders Institute for Brain, Cognition and Behaviour Radboud University Nijmegen Nijmegen The Netherlands

**Keywords:** minimal intervention principle, online feedback control, proprioception, sensorimotor control

## Abstract

Feedback corrections in reaching have been shown to be task‐dependent for proprioceptive, visual and vestibular perturbations, in line with predictions from optimal feedback control theory. Mechanical perturbations have been used to elicit proprioceptive errors, but have the drawback to actively alter the limb's trajectory, making it nontrivial to dissociate the subject's compensatory response from the perturbation itself. In contrast, muscle vibration provides an alternative tool to perturb the muscle afferents without changing the hands trajectory, inducing only changes in the estimated, but not the actual, limb position and velocity. Here, we investigate whether upper‐arm muscle vibration is sufficient to evoke task‐dependent feedback corrections during goal‐directed reaching to a narrow versus a wide target. Our main result is that for vibration of biceps and triceps, compensatory responses were down‐regulated for the wide compared to the narrow target. The earliest detectable difference between these target‐specific corrections is at about 100 ms, likely reflecting a task‐dependent feedback control policy rather than a voluntary response.

## 1 INTRODUCTION

Successful movement control requires the ability to rapidly adjust ongoing actions to account for changing conditions. Specifically, an efficient controller should only correct an ongoing movement if the desired outcome is under threat, either due to internal noise or an external perturbation. For example, when holding the rudder to steer a boat, unforeseen waves or gusts of wind should be corrected for more vigorously when navigating through a narrow channel compared to when at open sea.

Optimal feedback control (OFC) is a prominent theoretical framework to blend voluntary control and sophisticated, rapid corrections (Scott, 2004; Todorov & Jordan, 2002). In OFC, a goal‐directed movement unfolds from an optimal control policy that generates motor output based on ongoing state estimation. The state estimate is continuously updated based on sensory predictions from a forward model and signals from all observable sensors, respecting their delays (Crevecoeur, Munoz, & Scott, 2016; Crevecoeur & Scott, 2013). The control policy optimally trades between the constraints of the controlled plant and the task requirements, such as timing or spatial accuracy. As a result, an OFC controller will minimally correct for task‐irrelevant perturbations, referred to as the minimal intervention principle.

The minimal intervention principle has direct support from experiments that mechanically perturbed an ongoing reaching movement by exerting a force onto the limb by means of an exoskeleton (e.g., Nashed, Crevecoeur, & Scott, 2012; Pruszynski, Kurtzer, & Scott, 2008). Specifically, during the reach a step torque was applied around the elbow and/or shoulder, thereby changing the reach trajectory and inducing neural responses from various receptors along the limb, including afferents in the joints and stretched skin, tendons, and muscles. With different task constraints, such as different target shapes, the same perturbation elicits different corrective responses, which supposedly reflects the minimal intervention principle. In addition to mechanical perturbations, also corrections to visual (e.g., Franklin & Wolpert, 2008; Knill, Bondada, & Chhabra, 2011) and vestibular perturbations (Keyser, Medendorp, & Selen, 2017) have been shown to conform to the minimal intervention principle. Unlike mechanical perturbations, the latter two are purely sensory perturbations, changing only the estimated state of the arm without directly affecting the actual arm trajectory. In this study, we investigated the role of the proprioceptive system in task‐dependent feedback corrections, without actively altering the ongoing movement trajectory as done by mechanical step torques. To this end, we applied muscle vibration during an ongoing movement to change the estimated reach trajectory. Therefore, any observed change in motor output in response to vibration is exclusively attributable to a change in the internally estimated state of the limb.

Muscle vibration induces an illusory lengthening of a muscle, for example, producing a sensation of elbow extension when m. biceps brachii is vibrated and elbow flexion when m. triceps brachii is vibrated (Goodwin, McCloskey, & Matthews, 1972a). While the latter authors assessed effects of vibration on conscious perception, we will use the term “illusory” to denote a difference between estimated and actual limb state, while remaining neutral as to whether the estimated state is accessible to conscious perception. The main afferents that are influenced by muscle vibration are the primary and secondary muscle spindle endings, which contribute to the sense of limb movement and position, respectively, by signaling (changes of) muscle length (Burke, Hagbarth, Löfstedt, & Wallin, 1976a; Roll, Vedel, & Ribot, 1989; Vedel & Roll, 1982). Muscle spindle afferents are thought to contribute to the long‐latency (~50–100 ms) stretch response, as evoked by mechanical perturbations, which is the earliest component that exhibits the minimal intervention principle (for review, see Pruszynski & Scott, 2012). Such sophistication of rapid motor responses implies an intimate relationship with slower, voluntary control, as predicted by optimal feedback control (Scott, 2004). Thus, probing the proprioceptive system by muscle vibration offers an important alternative experimental avenue to validate the conclusions gained from mechanical perturbations.

Here, we investigate whether vibration‐evoked perturbations show task‐dependent features, conforming with the minimal intervention principle. Following the design of Nashed et al. (2012), subjects reached to a narrow or wide target, thereby defining different accuracy constraints for the reach endpoint in the direction orthogonal to the reach direction. Based on earlier findings for mechanical perturbations, we predict that corrective responses to vibration would be larger for the narrow than the wide target. Supposing that mechanical stretch perturbations and muscle vibration activate similar neural feedback loops, the task‐dependent modulation should become observable with a latency below 100 ms, that is, before any voluntary responses.

## 2 MATERIALS AND METHODS

### 2.1 Participants

Nine subjects (7 women, age 21–31 years) participated in the experiment after informed, written consent was obtained. All participants were right‐handed, had normal or corrected‐to‐normal vision and self‐reported to have no motor deficits. The total duration of the experiment was about 70–90 min, and all subjects were reimbursed for their time with 15 €. The study was part of a research program approved by the ethics committee of the Social Sciences faculty of the Radboud University in Nijmegen, The Netherlands, and conformed to the standards set by the Declaration of Helsinki, except for registration in a database.

### 2.2 Experimental setup

**Figure 1:**
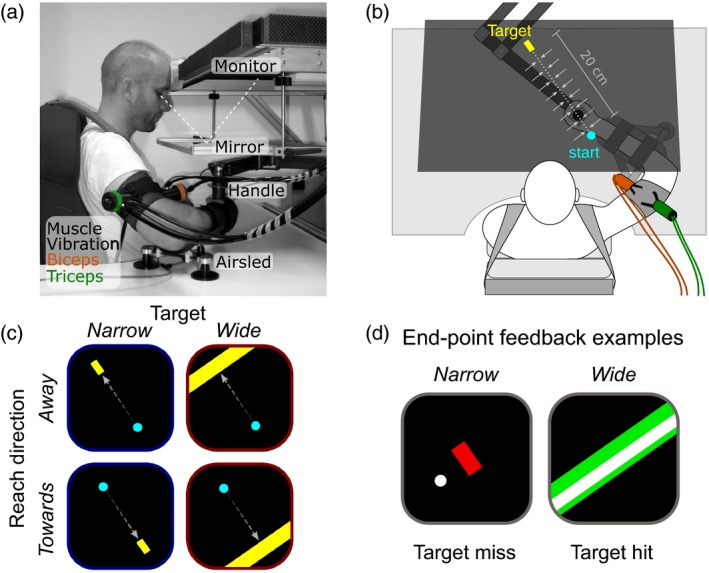
Experimental setup. (a) Subject holds the handle of a robotic manipulandum (vBOT). Visual stimuli are presented through a mirror. Pneumatic vibrators are attached to the biceps and triceps. (b) Top view of the setup, with the virtual image of the narrow target. Gray arrows indicate the mechanical channel that was active in 50% of trials. (c) Schematics of the two reach targets and reach directions. (d) Schematics of the end‐point feedback (white circle or rectangle), depending on target width. The target color changed from yellow to green if it was hit, or to red if it was missed. [Colour figure can be viewed at wileyonlinelibrary.com]

Subjects sat in a dimly lit room in front of a planar robotic manipulandum (vBOT; Howard, Ingram, & Wolpert, 2009), as shown in Figure 1a. Participants performed reaches in the horizontal plane with their right hand, while holding the handle of the manipulandum. Seat belts across the shoulders restrained trunk movements. Their reaching arm rested on an air sled floating on a glass top table, allowing almost frictionless movements. Visual stimuli were presented in the plane of movement via a semi‐silvered mirror, reflecting the display of an LCD monitor (model VG278H, Asus, Taiwan) suspended above. The display refresh rate was 120 Hz. All visual stimuli, that is, the start location of the reach, the target, and feedback about task performance were projected into the plane of movement. This virtual reality system covered the manipulandum, arm, and hand of the subject, thus preventing direct visual feedback of the arm. Subjects only received feedback about the end location of their reach as the cursor was extinguished throughout the reach. Handle forces were measured using a si*x*‐axis force transducer (model Nano 25, ATI Industrial Automation, Apex, NC, USA). Handle position and forces were monitored and stored at 1,000 Hz.

Vibration was applied using two pneumatic vibrators (model NTS 120HF, Netter, Germany) attached over the distal tendons of the m. biceps brachii and m. triceps brachii (Figure 1b). Custom, 3D‐printed cases were used to attach velcro straps to the vibrators. The casings were cylindrical, with a height of 7 cm and diameter of 4 cm, with a slightly convex base of 5 × 5.5 cm for easy attachment to the subjects’ arm. For attachment, a thin, elastic velcro band with one silicone side (facing the skin), were wrapped around the arm. The center of the vibrators was placed approximately 3–5 cm proximal to the olecranon. By adjusting the air pressure, both vibrators were set to a frequency of about 105 Hz and 1–2 mm amplitude — settings known to elicit illusory movement (Goodwin et al., 1972a). The setup's overall latency to start vibration was very reliable, with an overall *SD* < 2 ms. These values and settings were verified using a piezo element and an oscilloscope.

### 2.3 Experimental paradigm

Participants performed 20 cm reaching movements while holding the handle of the manipulandum (Figure 1a, b). Start and target positions of the reaches were arranged along an axis rotated 35° counter‐clockwise from straight‐ahead. To start a trial, participants positioned the handle (white circle of 0.6 cm diameter) at the starting position (cyan circle of 1.2 cm diameter). Then a target appeared (a yellow rectangle, Figure 1c) with a depth of 2 cm, and a width of either 1 or >30 cm, referred to as the narrow and wide target, respectively. Here, target depth denotes the radial dimension along the start‐target axis, and width denotes the lateral, perpendicular dimension with respect to that axis. The oblique reach direction was chosen to mainly require shoulder rotation for the radial direction and elbow torques for corrections along the target dimension that was manipulated. Start and target positions switched after each reach, and thus reach direction alternated between toward and away from the subject's body. The start and target positions were the same for all subjects. Relative to the subject's sternum the start position was about 25 cm in front and 5.7 cm to the right for reaches away from the subject, and about 41 cm in front and 5.7 cm to the left for reaches toward the subject. This resulted in a shoulder angle of about 50° and an elbow angle of about 90°. Target width and trial type were pseudo‐randomized between trials (see below).

After 1 s of waiting at the start position, a beep prompted the participant to initiate a reaching movement toward the target. Upon detection of a movement onset (handle speed > 5 cm/s), the cursor disappeared. If no movement was detected within 1 s, the trial was aborted and the participant received the instruction to “Move after the beep” and the trial was reset. The end position of a reach was defined as the first point where the handle speed was <5 cm/s.

The experiment contained four trial types. In free trials, movement was unconstrained and mainly served to reinforce the task and train subjects on the temporal and spatial constraints of the task (see below). In the other trial types, with vibration (biceps or triceps) or without vibration (null), movement was mechanically restricted by a mechanical channel that guided the reach toward the center of the target area. These mechanical channels enable quantification of corrective responses, in terms of force, to the vibration‐induced illusory limb displacements (see Data Analysis).

In free and null trials, feedback about reach accuracy was given for 200 ms, immediately after the reach ended (Figure 1d). In the narrow target condition, the reach endpoint was displayed as a circle of 0.6 cm diameter. In the wide target condition, performance feedback was only provided in the start‐target direction by displaying a thin line, parallel with the target's long edge, with a depth of 0.6 cm and width spanning the entire screen (>30 cm). This prevented subjects from gaining feedback about their lateral end position and forced them to only care about the reach accuracy in the radial dimension. If the reach ended within the target area, the target color changed from yellow to green, and if the target was missed, its color changed to red. For reaches with correct timing, an additional text was displayed. If the target was hit, the text stated “Great”, otherwise it stated “Move to Target”.

Participants were encouraged to reach the target within a certain time interval (700 ± 75 ms) by providing them with feedback about movement duration. If the reach took longer, participants heard a low‐pitched tone and saw the text “Too Slow” on the screen. Faster movements caused a high‐pitched tone to be played and the text “Too Fast” was shown. In trials with muscle vibration, no feedback about the end location of the reach was provided and only timing information provided. In free and null trials without muscle vibration, timing information was provided only if the target was hit, to emphasize the spatial constraint. These messages were introduced to promote similar movements across trials and participants, but failure to comply with these spatial and temporal constraints did not lead to exclusion of trials (see Data Analysis).

The experiment consisted of 600 trials. A break of at least 30 s was introduced after every set of 50 trials, to prevent fatigue. Vibration was applied in 25% of all trials, either to the m. biceps brachii (referred to as biceps trials) or the m. triceps brachii (triceps trials). The onset of the vibrators was triggered 100 ms after movement initiation (handle speed > 5 cm/s). Given their latency of about 50 ms (and overall *SD* < 2 ms), the actual vibration started about 150 ms after movement onset (specifically, 150 and 153 ms, see below). All vibration trials were executed in a mechanical force channel (stiffness: 5,000 N/m, damping: 2 N·s/m), simulated by spring‐like walls along a straight path from the start location of the hand toward the center of the target. The onset of the mechanical channel, and its start location, was also determined by the onset of the reaching movement. Vibrators and the mechanical channel were turned off at the end of movement (handle speed < 5 cm/s).

The remaining 75% of the trials were divided into trials without vibration and without mechanical channels (50% of all trials; free trials) and trials without vibration, but with force channels (25% of all trials; null trials). Trial types and target width (narrow vs. wide) were presented in pseudo‐random order: The randomization procedure ensured that before a given combination of reach direction and vibrated muscle was repeated, all other combinations were presented, and that between two vibration trials there were always 2–4 free or null trials. Prior to the experiment, subjects completed 50–150 training trials until they were confidently able to repeatedly adhere to the time and spatial requirements.

### 2.4 Data analysis

Analyses were performed with Python 3.6 (Python Software Foundation, RRID:SCR_008394, https://python.org), including packages h5py (The HDF Group, 1997), numpy (RRID:SCR_008633, van der Walt, Colbert, & Varoquaux, 2011), pandas (McKinney, 2010), matplotlib (RRID:SCR_008624, Hunter, 2007), and scipy (RRID:SCR_008058, Jones, Oliphant, & Peterson, 2001). Statistical tests were performed with R 3.4 (RRID:SCR_001905, R Core Team, 2017) via the rpy package, https://rpy2.bitbucket.io/. For repeated‐measures analyses of variance, we used R package ez 4.4 (Lawrence, 2016).

Although online feedback in the experiment was based on a 700 ± 75 ms window, we relaxed this criterion for the offline analyses. Trials were rejected from all analyses if their reach duration was outside the interval of 550–1,000 ms. This interval was chosen empirically to include at least 90% of trials per subject (mean 95.6%, range 91%–99%).

### 2.5 Preprocessing of force measurements

Our main analyses will focus on the influence of target width on the vibration induced forces exerted on the mechanical channels. To quantify the extent to which vibration evoked corrective responses, we calculated the component of the force that is perpendicular to the mechanical channel, derived from the force transducer. No filtering was applied to kinematic and force data, except to determine the average latency of the vibration setup (see below).

### 2.6 Onset latencies of vibration effects

To estimate the onset of vibration‐evoked responses, we determined the point at which the force traces from trials with muscle vibration started to diverge from trials without muscle vibration in the observed mean direction. For this, we used the segmented linear regression analysis from Weiler, Gribble, and Pruszynski (2015), who also provide their method's rationale, illustrations, and computer code.

In trials with muscle vibration, the vibrators were switched on 100 ms after reach onset. The pneumatic system of valves and tubes introduces a delay of the actual onset of vibration. The average delay was determined from the force traces collected in the experiment, using the onset detection method outlined above. To this end, the force traces were band‐pass filtered between 85 and 125 Hz using a third‐order Butterworth filter, to extract the vibrators’ frequency of about 105 Hz. The envelope of the band‐passed force measurement was determined using the absolute of its Hilbert transform. For detecting the onset delay of a vibrator on a specific muscle, we used the method from Weiler et al. (2015) and entered the pooled envelope traces from all participants’ null trials and the pooled envelope traces with biceps or triceps vibration, respectively. The search range for the delay was restricted between 100 and 200 ms after reach onset. Based on this, the latency of the vibrators on biceps and triceps were determined at 50 and 53 ms, respectively, resulting in onset of vibration from the onset of movement of 150 and 153 ms for the two vibration conditions. These time points served as the reference for the computation of onset times of subjects’ responses to vibration.

To estimate the onset times of subjects’ reactions, we used the conditions with compensation to vibration consistent with an illusory lengthening of the vibrated muscle, that is, biceps vibration for reaches away and triceps vibration for reaches toward the subject (see Results). We used the onset detection method by Weiler et al. (2015) and restricted it to consider only deviations in the direction of the observed group mean effects (see Results). To determine the onset times of responses to vibration, we entered the lateral forces of null trials versus trials with muscle vibration (biceps or triceps), separately for each target.

To determine the onset times of emerging differences between target conditions, we entered lateral forces of trials from the narrow versus wide condition, separately for each muscle. The search range for the onset was set between 150 and 550 ms after reach onset, corresponding with the physical onset of vibration and the duration of the shortest trials that were included in the analysis.

Due to the limited number of vibration trials within a given experimental condition (max. 18), we used a bootstrap analysis to arrive at stable onset estimates for each subject. We re‐calculated each AUC time series 1,000 times, after randomly choosing subsets of trials (with replacement), and re‐estimated the resulting onset with the method outlined above. The final onset estimate was the mean over bootstrap runs.

### 2.7 Statistical tests

We performed several repeated‐measures analyses of variance (RM‐ANOVA) on the group level, with the mean as measure of central tendency. The possible factors were target (levels: narrow, wide), reach direction (levels: toward, away), and vibrated muscle (levels: biceps, triceps). For the analysis of the lateral spread of the reach endpoints in the free trials, we performed a 2 × 2 RM‐ANOVA on the standard deviation of the endpoints with factors target width and reach direction. For each vibration condition we separately entered the lateral force differences from null trials at time point 550 ms into 2 × 2 RM‐ANOVAs with factors target width and reach direction. For radial endpoint differences between vibration and null trials we performed a 2 × 2 × 2 RM‐ANOVA with factors vibration, target width, and reach direction. For reach durations and peak speeds of null trials we computed a 2 x 2 RM‐ANOVA with factors target width and reach direction.

We used paired Wilcoxon signed‐rank tests to statistically compare response latencies to vibration and their difference between different target widths.

All tests were evaluated against a two‐tailed alternative hypothesis. We considered results as statistically significant at an alpha level of 0.05, although statistical outcomes close to this boundary can only be associated with weak evidence against the null hypothesis (Wetzels et al., 2011).

## 3 RESULTS

Right‐handed subjects were reaching toward or away from their body to either a narrow or a wide target, while holding the handle of a robotic manipulandum with their right hand. In 25% of trials, the reach was perturbed by applying muscle vibration over biceps or triceps during the reach. In these perturbation trials and in another 25% of trials without vibration, the hand was mechanically constrained to move in a straight line toward the target. In the remaining 50% of trials, neither vibration nor a mechanical channel was applied. Our main interest is whether feedback correction gains are categorically modulated between the narrow and wide target, which randomly changed from trial to trial. The minimal intervention principle predicts that the reduced spatial constraint in the wide target condition leads to reduced corrective responses (Todorov & Jordan, 2002).

### 3.1 Larger endpoint dispersion for the wide compared to the narrow target

If subjects were able to adjust their corrective feedback gains — based on target width — on a trial‐by‐trial basis, then naturally occurring variability during unperturbed and unconstrained reaches (free trials) should be allowed to accumulate along the task‐irrelevant dimension. For the wide target this task‐irrelevant dimension is perpendicular to the movement direction, and thus we expect larger perpendicular variability in reaches toward the wide target compared to the narrow target. We tested this prediction by entering the lateral standard deviations of reach endpoints in free trials into an RM‐ANOVA, which revealed a significant main effect of target width on the lateral spread of the endpoints (*F*_1,8_ = 40.4, *p *=* *0.0002; narrow: 0.6 cm (0.1 *SD*, range 0.5–0.8), wide: 0.9 cm (0.2 *SD*, range 0.7–1.1)). There was neither a significant effect of reach direction on lateral spread (*F*_1,8_ = 0.1, *p *=* *0.8), nor an interaction of reach direction and target width (*F*_1,8_ = 0.9, *p *=* *0.4). The increased spread of reach endpoints in the wide condition was seen in all participants, consistent with previous observations (Keyser et al., 2017; Nashed et al., 2012). We conclude that subjects were able to adapt their control policy in a task‐appropriate manner on a trial‐by‐trial basis.

### 3.2 Corrective responses to vibration are larger for the narrow compared to the wide target

**Figure 2:**
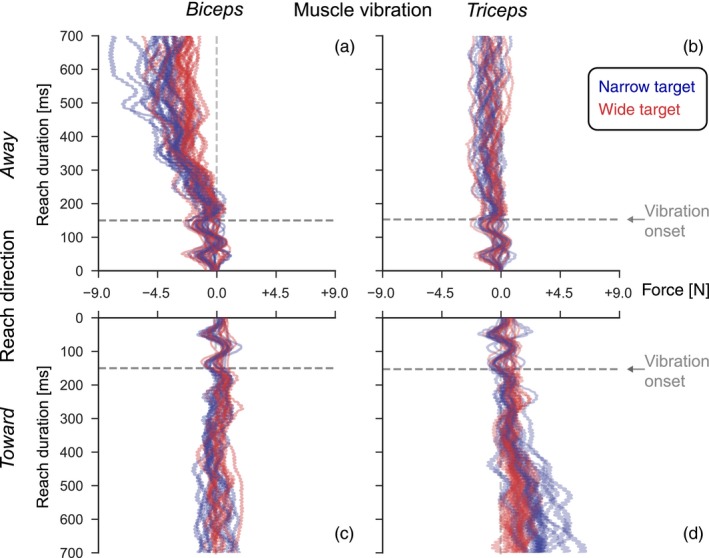
Lateral force data from an example subject. Each line represents the force generated in one trial. Negative and positive values on the x‐axis indicate counter‐clockwise and clockwise forces into the channel walls, respectively. The panel organization splits vibration condition and reach direction: Left column a, c: biceps vibration; right column b, d: triceps vibration; top row a, b shows reach direction away; bottom row c, d toward the subject. [Colour figure can be viewed at wileyonlinelibrary.com]

Our main interest concerned the target‐dependent feedback corrections to muscle vibration. The corrections were quantified in terms of the forces exerted onto a mechanical channel. Figure 2 shows the lateral force traces onto the mechanical channel for vibration trials of an example subject. The four panels show the separate conditions based on vibrated muscle and reach direction. The initial phase of reaches (until about 150 ms) exhibits a marked mechanical oscillation, resulting from the interplay of the subject's moving arm within the simulated channel walls. From about 150 ms, a high‐frequency oscillation can be discerned, indicating that the onset of the vibration can be directly picked up in the force traces. Vibration of biceps evoked compensatory forces in the elbow flexion direction, while triceps vibration evoked forces in the elbow extension direction. These responses are consistent with an illusory lengthening of the vibrated muscle and we interpret these forces as attempts to correct for an estimated perturbation of the movement trajectory. As Figure 2 shows, the compensatory forces are more pronounced for reaches to the narrow target (panels a, d). In addition, there is an interaction with reach direction, in that biceps vibration mainly affects reaches away from the subject, while triceps vibration mainly affects reaches toward the subject.

**Figure 3:**
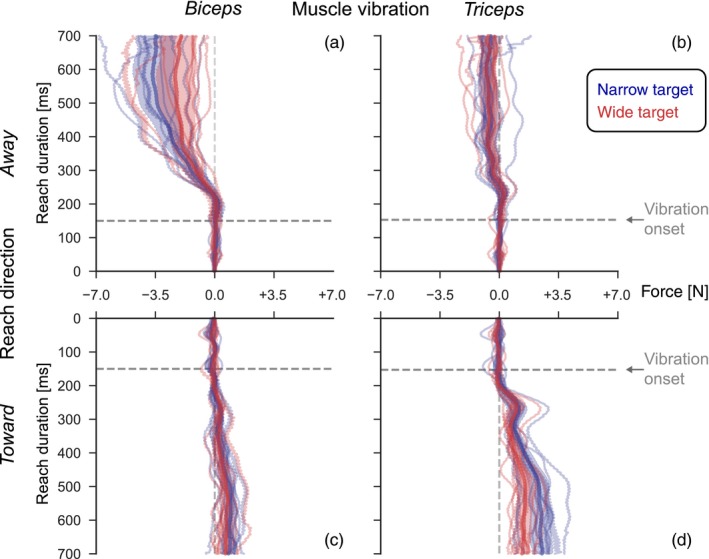
Effect of vibration. Group‐level comparison of vibration‐evoked lateral force corrections. Bold lines and shaded areas denote mean ±2 *SE* across subjects. Thin lines denote individual subject means. The panel organization (a, b, c, d) like in Figure 2. [Colour figure can be viewed at wileyonlinelibrary.com]

Figure 3 shows the vibration‐evoked and condition‐dependent corrections at the group level. Analogous to Figure 2, the four panels separate the conditions based on vibrated muscle and reach direction. To quantify the effects of vibration relative to null trials, the mean force trace in the null trials was subtracted from each trace with biceps or triceps vibration. This was done per subject and per experimental condition, that is, the four combinations of reach direction and target width. Thin lines indicate the mean responses for the individual subjects and the bold lines the mean response across subjects, with ±2 *SE* indicated by the shaded area. All combinations of reach direction and target width show force generation in response to muscle vibration of both biceps and triceps.

However, it is also clear from Figure 3 that biceps and triceps vibration do not result in opposite forces when applied during the same movement (away or toward). So, the compensation to vibration was not always consistent with an illusory lengthening of the vibrated muscle. When reaching away from the body, biceps vibration evoked compensatory forces that are consistent with elbow flexion in response to illusory lengthening of the biceps muscle. Similarly, when reaching toward the body, triceps vibration evoked forces consistent with elbow extension in response to illusory lengthening of the triceps muscle. In contrast, when reaching away from the body, triceps vibration induces a much smaller force compensation that is in the same direction as biceps vibration and when reaching toward the body, biceps vibration induces a much smaller force compensation in the same direction as triceps vibration.

**Figure 4:**
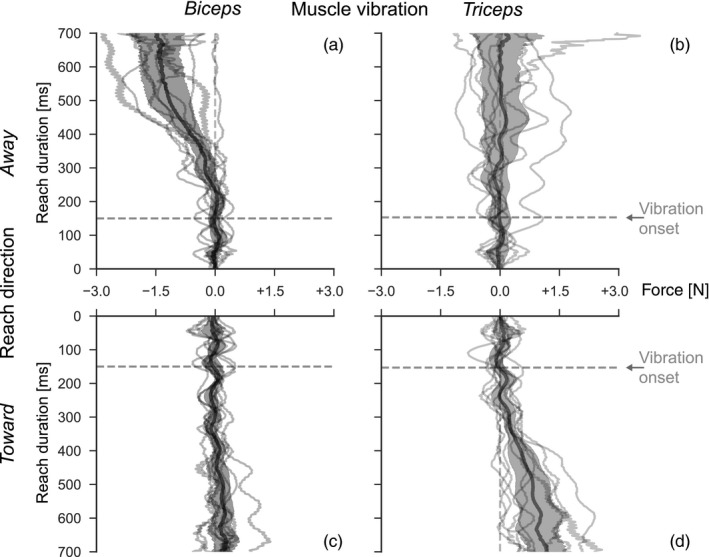
Effect of target width. Group‐level comparison of paired differences between force corrections in target conditions narrow ‐ wide. Bold lines and shaded areas denote mean ±2 *SE* across subjects. Thin lines denote individual subject means. The panel organization (a, b, c, d) like in Figures 2 and 3

To statistically compare the response magnitudes for the different conditions, we selected the compensatory forces at 550 ms after reach onset, that is, 400 ms after vibration onset. We tested at 550 ms because it is the latest time point without missing data from the shortest included trials; tests at 500 and 600 ms yield the same conclusions (data not shown) and the contrast between target widths is robust for earlier and later time points (see Figure 4). For each vibrated muscle (biceps and triceps), we performed an RM‐ANOVA. For biceps vibration, this revealed significant main effects of target width (*F*_1,8_ = 27.4, *p *=* *0.001) and reach direction (*F*_1,8_ = 41.8, *p *=* *0.0002), as well as a significant interaction (*F*_1,8_ = 14.0, *p *=* *0.006). Inspection of the means shows that the corrective responses to biceps vibration were larger for the narrow compared to the wide target condition (for reaches away, narrow: −3.6 N (1.6 *SD*, range −5.9 to −1.0) versus wide: −2.3 N (1.7 *SD*, range −5.3 to −0.3); for reaches toward, narrow: 0.9 N (0.5 *SD*, range 0.1–1.7) versus wide: 0.8 N (0.6 *SD*, range 0.1–1.9). For reach direction away, the average force decrease in the wide condition relative to the narrow condition was 63.0% (27.0 *SD*, range 12.5–98.4).

For the triceps vibration trials, we found a similar pattern, albeit reversed with respect to the reach direction. The RM‐ANOVA revealed a main effect of direction (*F*_1,8_ = 35.7, *p *=* *0.0003), and while the main effect of target remained nonsignificant (*F*_1,8_ = 5.1, *p *=* *0.05), their interaction was also significant (*F*_1,8_ = 10.5, *p *=* *0.01). Inspection of the means shows that for reaches toward the body the corrective responses were larger for the narrow compared to the wide target condition (narrow: 2.4 N (0.9 *SD*, range 1.1–4.0), wide: 1.5 N (0.7 *SD*, range 0.6–2.7)). For reaches away, the difference between target width was smaller and reversed in direction (narrow: −0.6 N (0.7 *SD*, range −1.4 to 1.0), wide: −0.7 N (0.8 *SD*, range −2.0 to 0.3)). For reaches toward, the average force decrease in the wide relative to the narrow condition was 66.1% (23.1 *SD*, range 27.7–95.7). Taken together, we conclude that for both muscles, corrections were indeed down‐regulated for the wide compared to the narrow target, contingent on reach direction. Corrections to biceps vibration were task‐modulated mainly in reaches away and less so in reaches toward the subject. Vibration of triceps revealed the reverse pattern; the force reduction to the wider target was only present in reaches toward the subject.

Given that we observed task‐dependent vibration effects relatively late in the reach, important questions are at what latency they start to appear and become different between target widths. To address these questions, we computed the temporal evolution of the difference between the compensatory forces for the narrow and wide target, per reach direction, vibrated muscle, and subject. Figure 4 shows the individual and average traces, again emphasizing the interaction between reach direction and vibrated muscle, with panels a and d showing a response that is modulated by the target width.

We investigated the onset latencies of vibration effects relative to the onset of physical vibration. The analyses were done in the conditions with robust compensation to vibration consistent with an illusory lengthening of the vibrated muscle, that is, biceps vibration for reaches away and triceps vibration for reaches toward the subject (see Figure 3). For biceps vibration during reaching away, the mean vibration‐evoked response onset was 62.7 ms (13.5 *SD*, range 49.0–94.4) for the narrow target and 61.2 ms (18.2 *SD*, range 33.4–89.4) for the wide target. There was no significant difference between target conditions (*V *=* *25, *p *=* *0.8), and the combined mean was 62.0 ms (13.1 *SD*, range 49.4–91.9). For triceps vibration during reaching toward, the mean onset was 34.8 ms (15.6 *SD*, range 8.9–61.8) for the narrow target and 45.8 ms (20.1 *SD*, range 26.7–90.0) for the wide target. There was also no significant difference (*V *=* *8, *p *=* *0.10), and the combined mean was 40.3 ms (16.1 *SD*, range 17.8–75.9).

We also investigated the onset times of target modulation in the conditions with significant amplitude difference of generated forces: biceps vibration during reaches away and triceps vibration during reaches toward the subject. For biceps vibration in direction away, mean onset was 105.7 ms (45.2 *SD*, range 56.2–168.2). For triceps vibration in direction toward, mean onset was 89.0 ms (52.4 *SD*, range 3.0–150.6). There was no significant difference between these conditions (*V *=* *27, *p *=* *0.7). The average mean onset across these subjects was 97.4 ms (28.7 *SD*, range 64.6–155.0).

### 3.3 Radial endpoints are affected by vibration but not by target width

The results above show that muscle vibration evokes compensatory forces into the channel walls, perpendicular to the reach direction, presumably due to an illusory change in muscle length and thus hand state. This illusory change in the current hand state might not only influence the perpendicular component of the reach direction, but also its radial component. Analogous to the analysis of force data, we focus on the effect of vibration on radial endpoints relative to the null condition, such that positive values indicate a reach ending further from the start than without vibration.

We entered the mean endpoint difference from null trials per subject and condition into a RM‐ANOVA, which revealed a significant intercept (*F*_1,8_ = 22.6, *p *=* *0.002), a significant main effect of reach direction (*F*_1,8_ = 16.9, *p *=* *0.003), and an interaction between reach direction and vibration condition (*F*_1,8_ = 26.6, *p *=* *0.0009). There were no significant main effects of target width (*F*_1,8_ = 0.007, *p *=* *0.9) or of vibration condition (*F*_1,8_ = 4.2, *p *=* *0.08). There were also no significant interactions between target width and reach direction (*F*_1,8_ = 0.3, *p *=* *0.6), target width and vibration condition (*F*_1,8_ = 0.003, *p *=* *1.0), nor between target width, reach direction, and vibration condition (*F*_1,8_ = 3.0, *p *=* *0.1). The significant, positive intercept shows that subjects had a general tendency to overshoot in vibration trials compared to null trials, across combinations of the vibrated muscle and reach direction. We inspected the means of the significant interaction between reach direction and vibration condition. Vibration of biceps is supposed to induce illusory elbow extension, whereas triceps vibration is supposed to result in illusory elbow flexion. This means that for reaches away from the body, biceps vibration should result in compensatory shortening of the reach compared to triceps vibration. For reaches toward the body, the compensatory movement in response to biceps vibration should result in lengthening of the reach compared to triceps vibration. Both predictions were confirmed by inspection of the means of the two vibrated muscles for the same reach direction. For reaches away from the body, mean endpoint overshoots relative to null trials were 0.3 cm (0.9 *SD*, range −1.3 to 1.3) for biceps vibration versus 1.0 cm (0.6 *SD*, range 0.0–1.7) for triceps vibration; that is, vibration of biceps evoked shorter reaches (less overshooting) than triceps vibration. In contrast, for reaches toward the subject, biceps vibration evoked longer reaches (more overshooting) than vibration of triceps: The mean overshoots were 2.0 cm (0.9 *SD*, range 0.8–3.7) for biceps vibration compared to 1.5 cm (1.1 *SD*, range −0.4 to 3.6) for triceps vibration. Taken together, this indicates that muscle vibration did not only affect subject's sense of movement, but also their estimated radial hand position for endpoint control. The lack of influence of target width on radial endpoints suggests that the task‐dependent effects on lateral force are indeed specific to the spatial target constraints.

### 3.4 Vibration effects are not explained by movement durations or maximum movement speeds

Our main result that vibration effects depend on target width and reach direction were found after subtracting the respective mean of null trials, and thus are independent of potential differences between these conditions. Nevertheless, we explored whether the reach durations or peak speeds in null trials were similar for the different conditions. For both variables, we computed an RM‐ANOVA. For reach durations, there was no main effect of target width (*F*_1,8_ = 4.6, *p *=* *0.06), no main effect of reach direction (*F*_1,8_ = 0.02, *p *=* *0.9), and no interaction (*F*_1,8_ = 1.5, *p *=* *0.3). The overall mean reach duration was 722.9 ms (55.6 *SD*, range 652.8–817.3). We also did not find significant differences among peak speeds of the reaches. Specifically, there was no main effect of target width (*F*_1,8_ = 1.4, *p *=* *0.3), no main effect of reach direction (*F*_1,8_ = 0.02, *p *=* *0.9), and no interaction (*F*_1,8_ = 0.001, *p *=* *1.0). The overall mean peak reach speed was 43.1 cm/s (3.6 *SD*, range 37.6–48.2). We conclude that neither differences in reach durations nor in maximum speeds in null trials can account for the observed effects of vibration; in particular we found no significant difference between the two target widths.

## 4 DISCUSSION

Our main finding is that the corrective reach responses to muscle vibration are task‐dependent: Corrective responses to the same physical perturbation were larger when reaching to the narrow compared to the wide target. The average onset of these corrective responses to vibration and the first noticeable difference between these responses for the different target widths were around 100 ms. Especially the early modulation of feedback responses by target width suggests that inputs from muscle afferents are part of a feedback control policy that takes into account the target‐specific task demands, in agreement with predictions from optimal feedback control theory (Todorov & Jordan, 2002).

### 4.1 Known properties of muscle spindles explain direction dependence in our results

Several features of our results have been reported previously, and may explain our observation that vibration effects depend on the reach direction. We found that biceps vibration was consistent with illusory elbow extension and thus corrective flexion only for reaches away from the subject, that is, when the biceps was lengthening (Figures 3 and 4). Also, the modulation by target width was only significant when reaching away (Figure 4). For triceps vibration, the dependency on reach direction was reversed, as would be expected from its role as the biceps’ antagonist; vibration effects and target modulation were stronger when reaching toward the subject, that is, when the triceps was lengthening. Previous findings offer an explanation for this interaction of muscle vibration and reach direction. Vibration applied to the muscle bellies or tendons of m. biceps brachii or m. triceps brachii induces involuntary contraction of the vibrated muscle in the passive limb (De Gail, Lance, & Neilson, 1966; Hagbarth & Eklund, 1966). Muscle vibration is accompanied by an illusory lengthening of the vibrated muscle, that is, with a mis‐perceived (change in) elbow angle (Goodwin, McCloskey, & Matthews, 1972b; Goodwin et al., 1972a). As we did not assess conscious perception in our experiment, we use the term “illusory” to denote a difference between the estimated and actual limb state, ignoring whether or not the estimated state results in a conscious percept. Vibration of multiple muscles induces complex multijoint illusory movement (Thyrion & Roll, 2010) that can be used as feedback to functionally improve control of prostheses even in re‐innervated muscles (Marasco et al., 2018). During voluntary movement, sensitivity to vibration is reduced in shortening muscles, and information about limb position and movement comes primarily from the lengthening muscle (Capaday & Cooke, 1981, 1983; Inglis & Frank, 1990; Inglis, Frank, & Inglis, 1991). Vibration mainly drives the muscle spindle afferents in relaxed muscle and additionally drives Golgi tendon organs during active contractions (Burke et al., 1976a; Fallon & Macefield, 2007; Roll et al., 1989; Vedel & Roll, 1982). Notably, especially the primary, group Ia muscle spindle afferents show greatly reduced activity when the muscle is shortening (Burke et al., 1976a; Roll et al., 1989; Vedel & Roll, 1982). During voluntary movement, the vibration‐induced activity masks the natural afferent activity (Roll et al., 1989). Together, the dependence of the muscle spindle afferents on the muscle state can account for the direction‐dependence of our results. We observed small but reliable responses to vibration of the contracting muscle that are not consistent with an estimated lengthening of this muscle. For example, biceps vibration evoked force into the left channel wall in reaches away (Figure 3a, b), but force into the right channel wall in reaches toward the body (Figure 3c, d). Supposing on one hand that a shortening muscle becomes essentially unresponsive to vibration, and on the other hand the vibration may spill over from the antagonist, one would expect this reversal. While we presume that in our setup the vibration stimulus was relatively focal, acting predominantly on biceps or triceps, we cannot exclude that some of the vibration spread to the antagonistic muscle. For example, the vibration might have been transmitted through the elastic velcro bands. Furthermore, the vibrators used here have a larger contact area (5 × 5.5 cm) than in other studies (3–3.5 cm diameter) (Capaday & Cooke, 1981; Goodwin et al., 1972a; Hagbarth & Eklund, 1966), possibly moving more tissue and thus introducing crosstalk between vibration sites. Finally, our setup of measuring lateral force into a mechanical channel during an ongoing reach might be more sensitive compared to classic kinematic measures, potentially explaining why this effect has not been reported before. However, for our main conclusion of task‐dependency, the exact placement of the vibrators or potential spread of their activity is irrelevant, because we compare corrections to different target widths that were randomized per trial and recorded in a single session, during reaches in the same direction. Due to the use of mechanical channels, the vibrated muscles were in similar length states across target conditions, thus excluding their states as alternative explanation for the task‐dependent differences.

### 4.2 Comparisons of task‐dependent reduction in corrections

Vibration‐evoked corrections were down‐regulated for reaches to the wide compared to the narrow target, for conditions in which vibration induced an illusory lengthening, that is, for biceps vibration in away reaches and triceps vibration in reaches toward the subject. The observed reduction in magnitude of the corrections by 34%–37% in the wide condition is similar to the 40% and 33% reduction found for visual and vestibular perturbations, respectively (Keyser et al., 2017; Knill et al., 2011). One might wonder why the corrections to the wide target were not abolished entirely. Two explanations for the remaining corrections have been put forward. First, it is possible that the task‐dependent modulation of the feedback gains overlap in time with faster, task‐independent responses, like the mono‐synaptic short‐latency responses for mechanical perturbations (Pruszynski, Kurtzer, & Scott, 2011). Second, Nashed et al. (2012) offer an explanation based on OFC, noting that a controller that trades off between accuracy and effort should also show corrections to a perturbation along a task‐irrelevant dimension to ultimately stabilize the hand at the target, which requires zero lateral velocity. Delaying this response would require more control commands later, hence it is optimal to respond immediately, albeit with appropriately reduced gain, even when reaching to the wide target. Further experiments are needed to dissociate these two hypotheses, for example, instructing subjects to reach through the target instead of stopping at it. In this case, the feedback responses should be further reduced under the second hypothesis, whereas they should remain unchanged under the first hypothesis.

### 4.3 Comparisons of onset timings in the literature

Sophisticated responses to stretch perturbations, also detected by the proprioceptive system, have been reported before (e.g., Nashed et al., 2012; Pruszynski et al., 2008; Selen, Shadlen, & Wolpert, 2012). However, all previous studies used mechanical perturbations that actively pushed the limb away from its ongoing trajectory. Here we also perturbed the proprioceptive system, but the vibration perturbation only induces an estimated error as opposed to the physical errors introduced by actual muscle stretch. One important outcome from experiments with stretch perturbations is that the long‐latency (~50–100 ms) stretch response already exhibits the minimal intervention principle, implying an intimate relation of rapid motor responses and voluntary control (Pruszynski & Scott, 2012; Scott, 2004).

Our own measurements imply a comparable latency for the target‐specific responses to vibration. However, previous studies have determined the latency of sophisticated responses based on electromyographic (EMG) recordings, whereas in this study we only have the latency in terms of the detected onset of compensatory forces. We found mean vibration‐evoked onsets of 62 and 40 ms for biceps and triceps, respectively. For eccentric contractions, the electromechanical delay between EMG and produced force was estimated at 33 ms (13 *SD*) for biceps and 30 ms (7 *SD*) for triceps (Norman & Komi, 1979). With this in mind, we interpret our observed onsets to fall into the short‐latency window, and as qualitatively consistent with EMG responses to vibration in flexor pollicis longus (Matthews, 1984), and the 60 ms previously reported for the biceps (Capaday & Cooke, 1983). We observed the mean task‐dependent modulation between target widths at 106 and 89 ms for biceps and triceps, consistent with medium‐ to long‐latency responses to mechanical perturbations (Nashed et al., 2012), thus confirming the expected early expression of the minimal intervention principle.

### 4.4 Neural processing of stretch and vibration may be different

The similarity of sophistication and timing between muscle vibration and previous findings from mechanical perturbations (in particular Nashed et al., 2012) begs the question whether they share the same neural circuitry, especially regarding their long‐latency response. Indeed, both types of perturbations are thought to be mainly mediated by the muscle spindle endings that signal (changes of) muscle length (Burke et al., 1976a; Pruszynski & Scott, 2012; Roll et al., 1989; Vedel & Roll, 1982). Furthermore, the task‐dependency observed during the long‐latency is thought to depend on a transcortical feedback pathway (Matthews, 1991; Omrani, Murnaghan, Pruszynski, & Scott, 2016; Pruszynski & Scott, 2012; Pruszynski, Kurtzer, Nashed et al., 2011; Scott, 2004). Notably however, stimulation by stretch or vibration are not identical. For example, vibration of flexor pollicis longus or flexor hallucis longus failed to elicit long‐latency responses although they were readily obtainable by stretch (Matthews, 1984; Matthews & Pickup, 1985). In other words, at least for certain muscles, their spindle afferent activity may lead to different (or no) motor responses, depending on whether they are elicited by stretch or by vibration. Incidentally, the medium‐ to long‐latency stretch response of flexor pollicis longus also fails to show task‐dependent modulation by verbal instruction, if corrected for background muscle activity (Capaday, Forget, & Milner, 1994). These discrepancies in responses to stretch and vibration for different muscles provides an experimental opportunity to test the generality of task‐dependency of the long‐latency response. As supported by the results from Thilmann, Schwarz, Töpper, Fellows, and Noth (1991), different mechanisms may underlie the long‐latency stretch reflex response at different joints.

### 4.5 Alternatives to interpreting task‐dependency as sensorimotor gain changes

We interpret our result of larger vibration‐evoked corrections for reaches to the narrow compared to the wide target as a task‐specific increase in the sensorimotor gain of the central nervous system's control policy. Although this is an interpretation on an algorithmic level and thus does not specify the physiological implementation, it suggests that before the perturbation, the sensorimotor periphery was in a similar state for both target conditions. In particular, muscle states may have been different, as subjects may have expressed higher levels of co‐contraction in the narrow compared to the wide target condition, as it has been argued that higher accuracy demands in goal‐directed movements result in increased muscular co‐contraction and joint stiffness to reduce the influence of neuro‐muscular noise on endpoint variability in the reach (Gribble, Mullin, Cothros, & Mattar, 2003; Selen, Beek, & van Dieën, 2006; Selen, Franklin, & Wolpert, 2009). It is also known that increased activity of a single elbow muscle increases the short‐latency response to stretch (e.g., Pruszynski, Kurtzer, Lillicrap, & Scott, 2009) and to vibration (Matthews, 1986). That result may extend to co‐contraction, as shown for the flexor and extensor pollicis longus of the thumb (Akazawa, Milner, & Stein, 1983). Based on the present results, we cannot exclude that task‐dependency has been mediated through the short‐latency responses’ “automatic gain scaling” to background loads. However, Nashed et al. (2012) found in a similar task that the elbow flexor's tonic muscle activity was only affecting the short‐latency but not the task‐modulated long‐latency response. In case gain‐scaling accounts for the task‐dependency, it would still constitute a sensorimotor gain change, albeit implemented peripherally, likely through the intrinsic organization of the spinal motoneuron pool (e.g., Matthews, 1986).

Another conceivable difference in peripheral states would be an increased proprioceptive gain in the narrow target condition, potentially also due to co‐contraction. With higher sensory gain, the same physical vibration might lead to more vigorous firing of the sensory neurons, thus leading to stronger responses without an increase in sensorimotor gain. A possible mechanism is fusimotor commands, that is, activation of gamma neurons to increase spindle sensitivity, as shown for isometric contractions (Vallbo, 1974). Indeed, isometric contraction of a muscle enhances the response of its spindle afferents to vibration (Burke, Hagbarth, Löfstedt, & Wallin, 1976b). However, co‐contraction should also lead to increased fusimotor drive in the antagonist, which contributes to movement coding of the joint (Ribot‐Ciscar & Roll, 1998), so it remains unclear whether it would lead to a net increase in sensitivity to vibration. Some evidence suggests that increased fusimotor drive does not necessarily translate into increased proprioceptive sensitivity (reviewed by Proske, Wise, & Gregory, 2000): For example, co‐contraction of elbow muscles reduced proprioceptive acuity in a movement detection task (Wise, Gregory, & Proske, 1998). This does not discard the possibility of sensory gain changes, since even in relaxed subjects, the fusimotor system seems to allow for task‐dependent control of muscle spindle feedback, e.g., through mental calculation or attention (Hospod, Aimonetti, Roll, & Ribot‐Ciscar, 2007; Ribot‐Ciscar, Rossi‐Durand, & Roll, 2000). Future research should explore whether such sensory gain modulation of the muscle spindles is used by the CNS for active movements, as studied here.

### 4.6 Opportunities of muscle vibration as experimental tool

To our knowledge, there are no other studies that quantified the effects of muscle vibration during a reaching movement in terms of evoked force into a simulated mechanical channel. We believe that our approach provides the necessary tools to study real‐time multisensory integration for online control, a topic that has been identified as an important avenue for further investigation (e.g., see Cluff, Crevecoeur, & Scott, 2015; Oostwoud Wijdenes & Medendorp, 2017; Scott, 2016). The fact that muscle vibration elicits an estimated rather than an actually altered limb state (with low onset variance < 2 ms) means that it can easily be combined with perturbations in other sensory modalities. For example, this could be exploited to study how the CNS deals with real‐time interaction between proprioception and vision in a straight‐forward manner, and thus contribute to the ongoing debate whether they are integrated into multimodal state estimation (Crevecoeur et al., 2016) or processed independently (Franklin, So, Osu, & Kawato, 2008; Oostwoud Wijdenes & Medendorp, 2017).

In summary, we show that proprioceptive perturbations induced by muscle vibration are sufficient to evoke task‐dependent feedback corrections during goal‐directed reaching. Like for mechanical perturbations of the limb, these target‐specific corrections started around 100 ms, likely reflecting a sophisticated feedback control policy.

## CONFLICT OF INTERESTS

The authors have no competing interests.

## AUTHOR CONTRIBUTIONS

JK, REFSR and LPJS conceived and designed research; REFSR collected data; JK and LPJS analyzed data; JK, REFSR, WPM, and LPJS interpreted data; JK prepared figures; JK drafted manuscript; JK, REFSR, WPM, and LPJS edited and revised manuscript. All authors have approved the final version of the manuscript and agree to be accountable for all aspects of the work. All persons designated as authors qualify for authorship, and all those who qualify for authorship are listed.

## Data Availability

The data and analysis code supporting this article can be accessed at the repository of the Donders Institute of Brain, Cognition, and Behaviour, https://data.donders.ru.nl. The persistent identifier is http://hdl.handle.net/11633/aaa6egmg.
