# Supplementary material for: Task‐dependent responses to muscle vibration during reaching
Source: Eur J Neurosci. 2018 Dec 11;49(11):1477–90. doi: 10.1111/ejn.14292 (PMC6618256; doi:10.1111/ejn.14292)
Supplement: Supplementary file 1 [file EJN-49-1477-s001.docx]

Task-dependent reach responses to muscle vibration

Keyser, Johannes ; Ramakers, Rob; Medendorp, W. Pieter; Selen, Luc

_____________________________________________________________________________________

| Review timeline: | Submission date: 01/06/18  Editorial Decision: Major Revision  Revision Received: 19/09/18  Editorial Decision: Minor Revision  Revision Received: 05/11/18  Accepted: 13/11/18 |  |
| --- | --- | --- |
|  |  |  |

Editor: Guillaume Rousselet

Reviewer 1: Konstantin Beinert

Reviewer 2: Eli Brenner

_____________________________________________________________________________________

1st Editorial Decision	14/07/18

_____________________________________________________________________________________

Task-dependent reach responses to vibration-induced illusions 

Dear Dr. Keyser, 

We have now received comments on your article from two experts. They are both overall positive but they have also highlighted a few issues that we would like you to address. In particular, some important information is missing and several areas require clarification. 

An important problem is the sample size: your experiment was clearly under-powered to detect interactions in a 2 x 2 x 2 design. Given the small number of participants but the relatively large number of trials, it would be more convincing to also report single-participant quantifications. For instance, you could report for each contrast how many participants show the group effect. For the group results, reporting SD across 9 participants is really not informative. Reporting min and max across participants  would be more useful - individual effect sizes even better. 

One reviewer questions the presence of conclusions amongst the results. From our editorial perspective, in the results section, it is fine to provide short interim conclusions to help the reader digest the information. 

Figures would be much easier to read if the axes were flipped, so that time is on the x-axis and comparisons can be assessed along the y-axis; it is much more common and easier to evaluate vertical differences. 

“Trials were rejected from all analyses if their reach duration was outside the interval of 550 to 1000 ms.” How was this window chosen? Can you confirm that truncating the response distributions does not introduce bias in your averages? Have you considered using robust measures of central tendency instead? 

Also justify similar sorts of parameters, such as “50 consecutive significant sample points (50 ms)”. See potential problems with this approach here: 
Piai, V., Dahlslatt, K. & Maris, E. (2014) Statistically comparing EEG/MEG waveforms through successive significant univariate tests: How bad can it be? Psychophysiology. 

“rejection of 26.2 (14.1 SD) trials across subjects” - do you mean “per” subject? 

“All tests were evaluated against a two-tailed alternative hypothesis, and were considered significant at an alpha level of 0.05.” - that’s fine but you need to mention that 0.05 can be associated with weak evidence against the null: 
Wetzels, R., Matzke, D., Lee, M.D., Rouder, J.N., Iverson, G.J. & Wagenmakers, E.J. (2011) Statistical Evidence in Experimental Psychology: An Empirical Comparison Using 855 t Tests. Perspectives on Psychological Science, 6, 291-298. 

Please also make the following changes: 
- Add figure legends. 
- add Research Resource Identifiers (RRIDs); 
- add an illustration of the technique to determine onset latencies + justifications of parameters (also mention that there is nothing special about the ROC analyses - it is exactly the same as using the Wilcoxon-Mann-Whitney U statistics divided by n1*n2 for instance; any robust measure of effect size for independent groups would work in fact); 
- mention the measure of central tendency used in the ANOVAs - presumably the mean; 
- report exact F and p values for all contrasts; 
- when reporting effect sizes, also report min, max, median across participants instead of SD - for instance in the sentence “On average, the force decrease in the wide condition relative to the narrow condition was 63.0% (27.0 SD).”; 
- rephrase “no difference” claims based on p values - that’s a frequentist fallacy - focus on effect sizes instead; 
- add reference number for data accessibility. 

When revising the manuscript, please embolden or underline major changes to the text so they are easily identifiable and DO NOT leave 'track change' formatting marks in your paper.  Please ensure that you provide a text and a figure file for the Graphical Abstract (as detailed in the instructions below).  When carrying out your revisions please refer to the checklist below and visit the EJN author guidelines at www.ejneuroscience.org 

When finalized, please upload your complete revised manuscript onto the website, as a Word file (.doc, or .docx).  Please also ensure that a complete set of tables and figures is included as separate files, even if these have not changed from the originals.  At this stage it is necessary to provide high resolution figures.  Please see important instructions below. 

Please go into https://mc.manuscriptcentral.com/ejn - Author Centre - manuscripts with decisions where you will find a 'create a revision' link under 'actions'. We ask that you please indicate the way in which you have responded to the points raised by the Editors and Reviewers in a letter. Please upload this response letter as a separate Word (.doc or PDF) file using the file designation "Authors' Response to Reviewers" when uploading your manuscript files.  Please DO NOT submit your revised manuscript as a new one.  Also, please note that only the  Author who submitted the original version of the manuscript should submit a revised version. 

If you are able to respond fully to the points raised, we would be pleased to receive a revision of your paper within 12 weeks. 

Thank you for submitting your work to EJN. 

Kind regards, 

John Foxe & Paul Bolam 
co-Editors in Chief, EJN 


Reviews: 

Reviewer: 1 

Comments to the Author 
Dear Editor, dear authors, 
thank you very much for the review invitation of "Task-dependent reach responses to vibration-induced illusions". The present experiment explores the effects of elbow muscle vibration on reaching performance in healthy participants. Although the experimental set up displays high technical effort, the title of the paper and the conducted experiments do not match and therefore questions the validity of the study. 
Misleading is in title is the term illusion. The term illusion should be removed! 
The result section and the discussion are far too long and should be shortened about 30%. 
Major 
The title is about vibration-induced illusion. In fact this is highly speculative because illusions were not assessed in this experiment. Please give information on how illusion was assessed in this experiment or change the title. 
Minor 
Introduction 
Information about illusion with respect to muscle vibration is misleading in this context. It should be removed. 
Method 
• How were participants recruited 
• Small sample size - how was it determined? 
• Please explain “corrected to normal vision” 
• How were “no motor deficits” assessed? 
Experimental set up 
Although animated pictures were provided for the reader, I have no really understanding how the setting was. I would suggest original pictures, with a sequence referring to the procedure will make the set up easier to understand for the reader 
• Please give joint degrees of the arm to better understand the resting position 
• CCW Please explain abbreviations before using 
• 600 Trials, please describe how this amount of trials was reached across the subjects during 60-90 minutes. How was fatigue assessed or prevented? 
The authors report about “illusory lengthening”. How was illusion assessed? I guess illusion was represented by a deviation of the reaching performance. 
Results 
Please remove explanations about statistical methods from the results section and take care to give all necessary information within the data analysis section. 
Please provide a table with the results for a better overview. 
Please remove any statements of the results section which compare present results of the experiment with other work and discuss this in the discussion section. Also avoid conclusions and interpretations ( i.e “These effects may reflect the muscles’ reduced responsiveness to vibration when shortening (e.g. Capaday & Cooke, 1981) in combination with spill-over of the vibration to the antagonist.) 
Discussion 
Avoid the term illusion and remove that part from the discussion because it is misleading for the reader. 
Further, the first part dealing with muscle spindle takes almost a half-page and adds nothing to the topic of interest. I recommend to remove it. 
“Sophisticated responses to proprioceptive perturbations have been reported before”. Somehow confusing for me because the reference frame for proprioceptive perturbation is missing. Please explain. 
It is stated that vibration caused only a perceptual error. Up to this moment this is speculative. 
The paragraph “Neural processing of stretch and vibration” is highly speculative and should be removed. 
What is meant by “higher proprioceptive sensitivity”. 


Reviewer: 2 

Comments to the Author 
I think this is a very interesting paper, but I think there are a few issues that need to be clarified or considered. The first is that the authors expect to find a response after between 50 and 100 ms. This makes sense for various reasons that are provided in the text. Indeed the response that depends on target width starts by about 250 ms (Figure 5), which is about 100 ms after the stimulation (probably slightly earlier). This is the main point of the paper. The authors spend a lot of time on some even earlier responses that may be present in Figure 3. I am not sure what I should make of these responses. Could they result from direct stimulation of the muscles when they are active? I found myself spending a lot of time contemplating figures 2-4. The mean onset of the response to triceps vibration of 32.5 ms raises doubts as to whether this could really be a response to sensory information. If the electromechanical delay given on page 23 is correct (30 ms for the triceps) this only gives 2.5 ms for sensory processing! This is rather short, even for a “short latency window” (page 23). The onset of target modulation is a much more reliable value, because it obviously cannot be an artifact of the method or a direct response to the stimulation. My feeling is therefore that it makes more sense to concentrate on this distinction, presenting the example in Figure 2 with narrow and wide targets indicated by colour and vibrations in separate panels. Figure 3 could then provide the same data at the group level, immediately showing the difference between wide and narrow (with muscles in separate panels). I see no need to directly compare the two muscles, and presenting all figures in the same format makes it all much easier to follow. Figure 4 is superfluous because what it shows can be seen in the other figures. Figure 5, that is now the main figure, may even also be superfluous because the difference may be clear from a regrouped figure 3, but it may add to the paper by showing the individual differences rather than the differences between the group data. 

An important issue in the analysis is estimating the onset of vibration-evoked responses (page 10). I get a sense of the method used, but I think the explanation should either be more complete, so that I can understand it without reading Weiler et al (2015), or else just refer to that method and give the values (if exactly that method is used). As it is, I am just left wondering whether fitting a 2-piece linear model is logical, and things like that. If the authors opt to explain their method maybe an illustration would help. 

On page 8 the authors mention a SD of the latency of the vibration of 2ms. Is this the overall SD? The mean of the values for individual participants? The SD of the mean values across participants? I assume the first because the participant should not matter, but it should be specified. I think it is now sort of specified that it is indeed the first option on pages 10 and 11, where the method of determining this is explained, but it is not very clear. It is better to combine these issues at one place and explain them more precisely. 

Later on page 8 the randomization is described. Target width was pseudo-random but blocked. From the text at the top of page 9 I would guess that target widths were blocked with the blocks presented in random order, but this is a guess. The authors should specify whether target widths were randomized or presented in separate blocks. 

I also have some suggestions about the presentation. I would try to group related issues a bit better. It took me a long time to try to determine whether the data were filtered. Data collection is described on page 6, but how the data were treated next appears on page 10. There the authors mention filtering between 85 and 125 Hz to determine the delay of the actual onset of vibration. Whether the data were also filtered to determine the onset of the response remains unclear. 

Apart from these issues I also have some very minor points 

On page 4 a part of a sentence is duplicated: “which contribute predominantly …” 

The last sentence of the main Data Analysis paragraph on page 9 ends with a sentence that I could not understand (“This is a conservative approach …”). I think some explanation is needed. 

The first part of the results section is a repetition of the procedure rather than a result. 

The “grand mean difference from zero” on page 18 is presumably referring to a main effect of vibration? If so, this terminology is not correct. 

The statement in the discussion (page 25) that the data may be explained by automatic gain scaling to background loads is not clear to me. How can this give an effect of target width? Do the authors just mean that the difference in response might be mediated by parameters such as muscle stiffness rather than the actual responses to the stimulation being different? Is this being contrasted with a “sophisticated feedback control policy” mentioned in the last sentence of the paper? Does this distinction matter? Why? 

------------------------ 

____________________________________________________________________________________

Authors’ Response	19/09/18

_____________________________________________________________________________________

# Authors' response to review comments

18–Sep–2018

We thank the editor and reviewers for the positive evaluation of our manuscript and the helpful comments and recommendations. Below we address all raised points, and underlined major changes to the text in the revised manuscript. Comments by the editor and reviewers’ comments are in *italic*.

*An important problem is the sample size: your experiment was clearly under-powered to detect interactions in a 2 x 2 x 2 design.*

It is important to note that we only present a single 2x2x2 RM-ANOVA on radial endpoints. This analysis is merely meant to complement the main results about lateral force (interactions with target width), which are based on 2 separate 2x2 RM-ANOVAs. In all these 2-level factor designs, interactions have the same degrees of freedom as the main effects. We did not find significant interactions with target width, which may indeed reflect a lack of power. However, irrespective of the (in)significance of these interactions, our main results and conclusion would not change. It would merely open up the question how specific the CNS distinguishes the target dimensions.

*Given the small number of participants but the relatively large number of trials, it would be more convincing to also report single-participant quantifications. For instance, you could report for each contrast how many participants show the group effect. For the group results, reporting SD across 9 participants is really not informative. Reporting min and max across participants would be more useful – individual effect sizes even better.*

We have added the ranges. Figures 3 and 4 now also contain single-subject quantifications of the main effects of interest (as thin lines, in addition to the group mean).

*One reviewer questions the presence of conclusions amongst the results. From our editorial perspective, in the results section, it is fine to provide short interim conclusions to help the reader digest the information.*

Done. (See details at the reviewer's comments.)

*Figures would be much easier to read if the axes were flipped, so that time is on the x-axis and comparisons can be assessed along the y-axis; it is much more common and easier to evaluate vertical differences.*

We purposely used a “birds-eye view” on the force traces, so that readers can geometrically understand the directions in which subjects respond to the vibration, since that depends on the vibrated muscle and reach direction (which is approximated by the direction of time on the vertical axis). To enable a balanced evaluation, we provide the requested versions of the figures with the axes flipped at the end of this document (note these are the revised figure versions in response to the suggestions from reviewer 2, see below). Unless the editors insist, we prefer to keep the intuitive link provided by the original figures.

*“Trials were rejected from all analyses if their reach duration was outside the interval of 550 to 1000 ms.” How was this window chosen? Can you confirm that truncating the response distributions does not introduce bias in your averages? Have you considered using robust measures of central tendency instead?*

We added the rationale to the text: “This interval was an empirical choice to include at least 90% of all trials per subject (mean 95.6%, range 91% – 99%)”. We chose this simple criterion to reject outlier trials that likely reflect non-representative lapses of attention or movement execution. The asymmetry of the interval around the instructed reach duration of 700 ms (–150 ms to +300 ms) is due to a longer tail in the distribution (see figure below). We re-analyzed the data without truncating the duration distributions and found very similar results, and arrive at the same conclusions, albeit several data samples became non-normally distributed due to outliers (significant Shapiro-Wilk tests). We conclude that in the present case, the mean is a sufficiently robust measure.

Figure: Histograms of all recorded reach durations. Colors indicate different subjects.

*Also justify similar sorts of parameters, such as “50 consecutive significant sample points (50 ms)”. See potential problems with this approach here:
Piai, V., Dahlslatt, K. & Maris, E. (2014) Statistically comparing EEG/MEG waveforms through successive significant univariate tests: How bad can it be? Psychophysiology.*

We acknowledge the arbitrariness of using 50 samples, and deleted the running Wilcoxon tests from the figures. Our intention behind them was to indicate that the exact time point of testing does not matter for the contrasts of interest (in the text we provide the analysis at t = 500 ms). Instead of repeated testing, we now plot ±2 SE around the group mean in figures 3 and 4, which roughly corresponds with the 95% CI of the mean. Plotting the mean ±2 SE serves the same purpose (but now using descriptive statistics); in fact, the time intervals where zero is excluded from the shaded area are overlapping almost exactly where the running Wilcoxon tests are significant.

*“rejection of 26.2 (14.1 SD) trials across subjects” - do you mean “per” subject?*

Correct. We rectified the text.

*“All tests were evaluated against a two-tailed alternative hypothesis, and were considered significant at an alpha level of 0.05.” – that’s fine but you need to mention that 0.05 can be associated with weak evidence against the null:
Wetzels, R., Matzke, D., Lee, M.D., Rouder, J.N., Iverson, G.J. & Wagenmakers, E.J. (2011) Statistical Evidence in Experimental Psychology: An Empirical Comparison Using 855 t Tests. Perspectives on Psychological Science, 6, 291-298.*

We added this precaution to the text: “We considered results as statistical significant at an alpha level of 0.05, although statistical outcomes close to this boundary can only be associated with weak evidence against the null hypothesis (Wetzels et al., 2011).”

*Please also make the following changes:*

- *Add figure legends.*

  We have updated the figures and legends following comments of the editor and reviewers.
- *add Research Resource Identifiers (RRIDs);*

  Done, we added the RRIDs for the software packages we used.
- *add an illustration of the technique to determine onset latencies + justifications of parameters (also mention that there is nothing special about the ROC analyses - it is exactly the same as using the Wilcoxon-Mann-Whitney U statistics divided by n1*n2 for instance; any robust measure of effect size for independent groups would work in fact);*

  We refer to the illustration provided by the original authors of the technique (Weiler et al., 2015), who also point out that there is no special status about using ROC (p. 3244). We use the same parameters as used by these authors, apart from the reported search ranges and restriction to be one-sided (see below). We removed our abbreviated explanation from the Methods, and instead only refer to the original authors in the text: “For this, we used the segmented linear regression analysis from Weiler et al. (2015), who also provide their method's rationale, illustrations, and computer code.” We also removed the reference to the Method in the Results section.
- *mention the measure of central tendency used in the ANOVAs – presumably the mean;*

  Done. We used the mean, consistent with our report of means.
- *report exact F and p values for all contrasts;*

  Done.
- *when reporting effect sizes, also report min, max, median across participants instead of SD – for instance in the sentence “On average, the force decrease in the wide condition relative to the narrow condition was 63.0% (27.0 SD).”;*

  We report effect sizes for ANOVAs and added ranges across participants (min and max). Instead of the median, we keep the mean because it is also used in the ANOVAs. We also computed Shapiro Wilk tests for every post-hoc *t*-test and found that none were significant at an alpha-level of 0.05.
- *rephrase “no difference” claims based on p values – that’s a frequentist fallacy – focus on effect sizes instead;*

  Done. We rephrased all such instances in the text to “no significant difference”.
- *add reference number for data accessibility.* The policy of our institute's repository ([https://data.donders.ru.nl](https://data.donders.ru.nl/)) requires acceptance of an article before a permanent, immutable reference becomes available. In that case, we will immediately update the manuscript. For review purposes, a temporary access URL can be made available upon request.

## Reviewer: 1

*Comments to the Author*

*Dear Editor, dear authors,*

*thank you very much for the review invitation of "Task-dependent reach responses to vibration-induced illusions". The present experiment explores the effects of elbow muscle vibration on reaching performance in healthy participants. Although the experimental set up displays high technical effort, the title of the paper and the conducted experiments do not match and therefore questions the validity of the study.*

*Misleading is in title is the term illusion. The term illusion should be removed!*

*The result section and the discussion are far too long and should be shortened about 30%.*

### Major

*The title is about vibration-induced illusion. In fact this is highly speculative because illusions were not assessed in this experiment. Please give information on how illusion was assessed in this experiment or change the title.*

We concede that the term “illusion” may be misleading to readers for who this implies conscious perception and would therefore expect this to be assessed directly as such. In this sense, the referee is correct in that we did not assess (conscious) illusions in our experiment. For the present manuscript, we deem it irrelevant whether vibration induced conscious perception and choose to remain in a neutral position. To avoid possible confusion, we changed the title to “Task-dependent reach responses to muscle vibration” and removed the term from the abstract: “In contrast, muscle vibration provides an alternative tool to perturb the muscle afferents without changing the hands trajectory, inducing only ~~illusory~~ changes in the estimated, but not the actual, limb position and velocity”. Further, we added a clarifying sentence on how we interpret the term “illusory” at its first occurrence in the Introduction and Discussion (see replies below) and changed any occurrence of “perceptual” to “estimated”/“illusory” throughout the text to avoid the connotation of perception with consciousness.

### Minor

# Introduction

*Information about illusion with respect to muscle vibration is misleading in this context. It should be removed.*

Instead of removing the term, we prefer to keep the phrasing of the cited research that directly showed that vibration-induced illusions can be available to conscious perception (Goodwin et al., 1972a). We clarified this potential source of confusion at the first occurrence of the term in the revised manuscript: “Muscle vibration induces an illusory lengthening of a muscle, for example producing a sensation of elbow extension when m. biceps brachii is vibrated and elbow flexion when m. triceps brachii is vibrated (Goodwin et al, 1972a). While the latter authors assessed effects of vibration on conscious perception, we will use the term 'illusory' to denote a difference between estimated and actual limb state, while remaining neutral as to whether the estimated state is accessible to conscious perception.”

# Method

- *How were participants recruited.*

  We recruited students from our university.
- *Small sample size – how was it determined?*

  We based our sample size of N = 9 on a similar experiment by Nashed et. al (2012), specifically their experiment 1: the authors manipulated target width and mechanically perturbed the subject's actual hand position. Their sample sizes were N = 10 when target size was blocked and N = 8 in a second version in which target size was randomized, like in our study.
- *Please explain “corrected to normal vision”.*

  This is standard jargon and indicates that subjects wore contact lenses or glasses, if needed.
- *How were “no motor deficits” assessed?*

  This was based on a self-report; we changed the phrasing to “All participants […] self-reported to have no motor deficits.”

# Experimental set up

*Although animated pictures were provided for the reader, I have no really understanding how the setting was. I would suggest original pictures, with a sequence referring to the procedure will make the set up easier to understand for the reader.*

Done. We replaced figure 1a with an annotated photograph to clarify the setup.

- *Please give joint degrees of the arm to better understand the resting position.*

  We added the following text: “The start and target positions were the same for all subjects. Relative to the subject's sternum the start position was about 25 cm in front and 5.7 cm to the right for reaches away from the subject, and about 41 cm in front and 5.7 cm to the left for reaches toward the subject. This resulted in a shoulder angle of about 50 degrees and an elbow angle of about 90 degrees.”
- *CCW Please explain abbreviations before using.*

Done. Replaced with “counter-clockwise”.

- *600 Trials, please describe how this amount of trials was reached across the subjects during 60-90 minutes. How was fatigue assessed or prevented?*

  Physical fatigue is hardly a concern in this type of experiment and setup. The arm is supported by an airsled, taking away the need to lift the arm against gravity and minimizing the required forces to move the arm (see Methods and figures 1 a,b). Mental fatigue was prevented by breaks after every 50 trials (see Methods). We corrected the previous break duration and added the rationale for the breaks: “A break of at least ~~one minute~~ 30 s was introduced after every set of 50 trials, to prevent fatigue”. Hence, the total break duration was at least 11*30 = 5.5 minutes. Including these breaks, the experiment duration for all 600 trials was on average 35.2 minutes (range 33.1 – 39.8 minutes). The rest of the 60-90 minutes were used to obtain informed consent and explanation of the experiment and training of the task.

*The authors report about “illusory lengthening”. How was illusion assessed? I guess illusion was represented by a deviation of the reaching performance.*

Done, see above. We clarified in the text that our use of “illusory” remains neutral to whether this is consciously perceived.

# Results

*Please remove explanations about statistical methods from the results section and take care to give all necessary information within the data analysis section.*

Done.

*Please provide a table with the results for a better overview.*

Done.

*Please remove any statements of the results section which compare present results of the experiment with other work and discuss this in the discussion section.*

There was only one such statement in the Results: “The increased spread of reach endpoints in the wide condition was seen in all participants, consistent with previous observations (Nashed, Crevecoeur, and Scott 2012; Keyser, Medendorp, and Selen 2017).” We prefer to keep as-is, since it links to very similar experiments but is not relevant enough to be picked up in the discussion.

*Also avoid conclusions and interpretations (i.e “These effects may reflect the muscles’ reduced responsiveness to vibration when shortening (e.g. Capaday & Cooke, 1981) in combination with spill-over of the vibration to the antagonist.)*

Done, removed.

# Discussion

*Avoid the term illusion and remove that part from the discussion because it is misleading for the reader.*

See also above. To avoid misleading the reader, we clarified the distinction between interpretation of our results and citing previous literature about consciously perceived illusions: “Since we did not assess conscious perception in our experiment, we use the term 'illusory' to denote a difference between the estimated and actual limb state, ignoring whether or not the estimated state results in a conscious percept.”

*Further, the first part dealing with muscle spindle takes almost a half-page and adds nothing to the topic of interest. I recommend to remove it.*

We respectfully disagree. This paragraph provides the necessary context for our interpretation of the effects of reach direction.

*“Sophisticated responses to proprioceptive perturbations have been reported before”. Somehow confusing for me because the reference frame for proprioceptive perturbation is missing. Please explain.*

Done, we now write: “Sophisticated responses to stretch perturbations, also detected by the proprioceptive system, have been reported before”.

*It is stated that vibration caused only a perceptual error. Up to this moment this is speculative.*

Clarified. We re-formulated to “estimated error”.

*The paragraph “Neural processing of stretch and vibration” is highly speculative and should be removed.*

We agree that there is some speculation in this paragraph, which is meant to put our results into a broader perspective and suggest new avenues of research. We prefer to give that guidance to the interested readers.

*What is meant by “higher proprioceptive sensitivity”.*

We meant this to be synonymous with the proprioceptive system's gain (to transduce physical vibration into neural activity), and clarified this in the respective sentence: “Another conceivable difference in peripheral states would be an increased proprioceptive ~~sensitivity~~ gain in the narrow target condition, potentially also due to co-contraction”.

## Reviewer: 2

*Comments to the Author*

*I think this is a very interesting paper, but I think there are a few issues that need to be clarified or considered. The first is that the authors expect to find a response after between 50 and 100 ms. This makes sense for various reasons that are provided in the text. Indeed the response that depends on target width starts by about 250 ms (Figure 5), which is about 100 ms after the stimulation (probably slightly earlier). This is the main point of the paper. The authors spend a lot of time on some even earlier responses that may be present in Figure 3. I am not sure what I should make of these responses. Could they result from direct stimulation of the muscles when they are active?*

The earlier responses are important to enable comparisons with previous findings on the responses to vibration as such, and to provide context for the later onset of target-dependent differences. For both, there exist relatively strong expectations based on previous literature on mechanical perturbations. Note that we revised all onset estimates (see next reply below), but without need to change our interpretations: For the difference between target widths, the mean onset estimates were revised by +26.5 ms for biceps and +19.6 ms for triceps. We do not think the earlier responses to vibration can be attributed to direct stimulation of the muscles, as such an effect was never described in the previous vibration literature (with very similar settings), and the onsets should be much earlier and more consistent.

*I found myself spending a lot of time contemplating figures 2-4. The mean onset of the response to triceps vibration of 32.5 ms raises doubts as to whether this could really be a response to sensory information. If the electromechanical delay given on page 23 is correct (30 ms for the triceps) this only gives 2.5 ms for sensory processing! This is rather short, even for a “short latency window” (page 23). The onset of target modulation is a much more reliable value, because it obviously cannot be an artifact of the method or a direct response to the stimulation.*

We are fully aware of this apparent discrepancy, and prefer to share rather than hide it. We attribute it to the inherent uncertainty in onset estimation and to differences in methods of previous papers. Prompted by this comment, we scrutinized all analysis steps within the onset estimation pipeline and revised one parameter in Weiler et al.'s (2015) method to further avoid very early onset estimates that are likely false positives: Instead of considering deviations from change in either direction as significant (ROC AUC 0.5±0.25), we set it to be one-sided (0.5–0.25 or 0.5+0.25) depending on the observed effect. To reflect this in the method section, we added “We used the onset detection method by Weiler et al. (2015) and restricted it to consider only deviations in the direction of the observed group mean effects (see Results).” This change results in fewer false-positives. For responses to vibration, this adjustment resulted in mean onset estimates that were up to +12.2 ms later.
Even after this change there remains considerable uncertainty in our mean onset measurements (similar to the spread in estimates in figure 8B from Weiler et al., 2015). The reviewer's quoted result of 32.5 ms for triceps vibration came from the narrow target condition that was revised to 34.8 ms (15.6 SD). The onset latencies for the wide target condition appear higher, at 45.8 ms (20.1 SD). Since there is no (reason to suspect a) significant latency difference between both target conditions, we consider the combined mean of 40.3 ms (16.1 SD, N=9), with a 95% CI from 27.9 to 52.7 ms. The values reported by Norman & Komi (1979) for the electromechanical delay entail a 95% CI from 28 to 32 ms (mean 30 ms, 7 SD, N=36). Subtracting this from our combined estimate yields a 95% CI for the mean difference from 4.4 to 16.2 ms, with the upper limit approaching realistic sensory processing time for a putative spinal mechanism.

*My feeling is therefore that it makes more sense to concentrate on this distinction [target widths], presenting the example in Figure 2 with narrow and wide targets indicated by colour and vibrations in separate panels. Figure 3 could then provide the same data at the group level, immediately showing the difference between wide and narrow (with muscles in separate panels). I see no need to directly compare the two muscles, and presenting all figures in the same format makes it all much easier to follow. Figure 4 is superfluous because what it shows can be seen in the other figures.*

We agree, followed the advice for figures 2 and 3, and removed figure 4. We adapted the text to reflect this change in layout. Note that in figure 2, we no longer show null trials for visual clarity.

*Figure 5, that is now the main figure, may even also be superfluous because the difference may be clear from a regrouped figure 3, but it may add to the paper by showing the individual differences rather than the differences between the group data.*

We agree with the reasoning and keep figure 5 (now figure 4).

*An important issue in the analysis is estimating the onset of vibration-evoked responses (page 10). I get a sense of the method used, but I think the explanation should either be more complete, so that I can understand it without reading Weiler et al (2015), or else just refer to that method and give the values (if exactly that method is used). As it is, I am just left wondering whether fitting a 2-piece linear model is logical, and things like that. If the authors opt to explain their method maybe an illustration would help.*

Done. Since we did not modify the method, we opted to refer to Weiler et al. without further explanation: “For this, we used the segmented linear regression analysis from Weiler et al. (2015), who also provide their method's rationale, illustrations, and computer code.” We already reported the search ranges for onset detection for the different applications in the manuscript: “The search range for the delay was restricted between 100 and 200 ms after reach onset.” and “The search range for the onset was set between 150-550 ms after reach onset, corresponding with the physical onset of vibration and the duration of the shortest trials that were included in the analysis.”

*On page 8 the authors mention a SD of the latency of the vibration of 2 ms. Is this the overall SD? The mean of the values for individual participants? The SD of the mean values across participants? I assume the first because the participant should not matter, but it should be specified. I think it is now sort of specified that it is indeed the first option on pages 10 and 11, where the method of determining this is explained, but it is not very clear. It is better to combine these issues at one place and explain them more precisely.*

Clarified. It refers to the overall variability in latency, independent of participants. We added to the setup description: “The setup's overall latency to start vibration was very reliable, with an overall SD < 2 ms. These values and settings were verified using a piezo element and an oscilloscope.” The mean latency of the vibration after reach onset was determined from the participant data, and the method is described later in the Data Analysis section for this reason.

*Later on page 8 the randomization is described. Target width was pseudo-random but blocked. From the text at the top of page 9 I would guess that target widths were blocked with the blocks presented in random order, but this is a guess. The authors should specify whether target widths were randomized or presented in separate blocks.*

Target widths were not presented in trial blocks but varied on a trial-to-trial basis. We admit our formulation about “blocks” was misleading; they were meant to refer to sets of trials that contained every combination of trial type and target width. We revised the text as follows: “Trial types and target width (narrow vs. wide) were ~~applied pseudo-randomly in a blocked fashion~~ presented in pseudo-random order: The randomization procedure ensured that ~~each~~ before a given combination of reach direction and vibrated muscle was ~~applied within a block of trials~~ repeated, all other combinations were presented, and that between two vibration trials there were always 2-4 free or null trials.”

*I also have some suggestions about the presentation. I would try to group related issues a bit better. It took me a long time to try to determine whether the data were filtered. Data collection is described on page 6, but how the data were treated next appears on page 10. There the authors mention filtering between 85 and 125 Hz to determine the delay of the actual onset of vibration. Whether the data were also filtered to determine the onset of the response remains unclear.*

Done. We clarified this in the revised section on pre-processing: “No filtering was applied to kinematic and force data, except to determine the average latency of the vibration setup (see below).”

# Apart from these issues I also have some very minor points

*On page 4 a part of a sentence is duplicated: “which contribute predominantly …”*

Rectified.

*The last sentence of the main Data Analysis paragraph on page 9 ends with a sentence that I could not understand (“This is a conservative approach …”). I think some explanation is needed.*

We removed the sentence in question (“This is a conservative approach because the subsequent analyses include more variation between trials”). It was originally meant in reference to the relatively long interval for trial inclusion based on trial duration (700–150 ms to 700+300 ms), and the rationale has been clarified in the text now: “This interval was an empirical choice to include at least 90% of all trials per subject (mean 95.6%, range 91% – 99%)”. See also our reply above on how this window was chosen.

*The first part of the results section is a repetition of the procedure rather than a result.*

Correct, we repeat some key aspects of the methods to ease reading. We leave it at the editor's discretion whether it should be deleted.

*The “grand mean difference from zero” on page 18 is presumably referring to a main effect of vibration? If so, this terminology is not correct.*

It is not referring to a main effect, but the overall mean of the data set (which is composed of differences from null trials). We renamed it to “intercept” for clarification (see also table 4): “The significant, positive intercept shows that subjects had a general tendency to overshoot in vibration trials compared to null trials, across combinations of the vibrated muscle and reach direction.”

*The statement in the discussion (page 25) that the data may be explained by automatic gain scaling to background loads is not clear to me. How can this give an effect of target width? Do the authors just mean that the difference in response might be mediated by parameters such as muscle stiffness rather than the actual responses to the stimulation being different? Is this being contrasted with a “sophisticated feedback control policy” mentioned in the last sentence of the paper? Does this distinction matter? Why?*

The reviewer's interpretation regarding a possible mediation via muscle stiffness is correct. However, this is meant to be a possible physiological implementation, not in direct contrast with a sophisticated feedback control policy. To clarify this in the text, we changed in the text: “Although this is an interpretation on an algorithmic level and thus does not specify the physiological implementation […]”.

## Alternative figure versions (as requested by the editor)

*Figures would be much easier to read if the axes were flipped, so that time is on the x-axis and comparisons can be assessed along the y-axis; it is much more common and easier to evaluate vertical differences.*

In our figures we purposely use a “birds-eye view” on the force traces, so that readers can geometrically understand the directions in which subjects respond to the vibration, since that depends on the vibrated muscle and reach direction (which is approximated by the direction of time on the vertical axis). To enable a balanced evaluation, we provide the requested versions of the figures with the axes flipped below (note these are the revised figure versions in response to the suggestions from reviewer 2, see above). Unless the editors insist, we prefer to keep the intuitive link provided by the original figures.

Alternative figure 2.

Alternative figure 3.

Alternative figure 4.

2^nd^ Editorial Decision	01/11/18

_____________________________________________________________________________________

ask-dependent reach responses to muscle vibration 

Dear Dr. Keyser, 

Your revised manuscript has been re-evaluated by one of the external reviewers as well as by the Editorial Team.  We are pleased to inform you that we expect that it will be accepted for publication in EJN after you have dealt with the few minor points raised by the reviewer. 

As you can see below, although the reviewer considers that the paper is much improved, he/she continues to raise a few points that will need to be addressed in a final revised version.  We are in agreement with the reviewer about replication of the data in the tables; if it is reported in the text then it is not necessary to tabulate it.  Please also ensure that your reporting of statistical analyses are completely transparent and accessible.  Please carefully each pint that the reviewer has raise in your revised version. 

Please include an abbreviation list in the paper. 

When finalised, please upload your complete revised manuscript onto the website as a Word (.doc or .docx), or .rtf file.  Please also ensure that a complete set of tables and figures is included as separate files, even if these have not changed from the originals.  At this stage it is necessary to provide high resolution figures.  Please see important instructions below. 

Please go into https://mc.manuscriptcentral.com/ejn - Author Centre - manuscripts with decisions where you will find a 'create a revision' link under 'actions'. We ask that you please indicate the way in which you have responded to the points raised by the Editors and Reviewers in a letter. Please upload this response letter as a separate Word (.doc) file using the file designation "Authors' Response to Reviewers" when uploading your manuscript files. Please DO NOT submit your revised manuscript as a new one.  Also, please note that only the  Author who submitted the original version of the manuscript should submit a revised version. 

If you are able to respond fully to the points raised, we shall be pleased to receive a revision of your paper within 30 days. 

Thank you for submitting your work to EJN. 

Best wishes, 

Paul Bolam & John Foxe 
co-Editors in Chief, EJN 

Reviews: 

Reviewer: 2 

Comments to the Author 
I think this is a good revision. I have a few suggestions but none of them are very important. 

1. I would change the title again. It now sounds as if participants are reaching to the muscle vibration. Maybe remove the word ‘reach’. Just “Task-dependent responses to muscle vibration” does not change the intention, I think. 

2. I would remove all the tables. Except for the fact that all the important statistics are also presented in the text (it is up to the editor to decide whether such duplication is acceptable) I think having all these tables emphasizes the sloppy way in which statistical hypothesis testing is currently used in our field (in this and many other studies). On page 19, just before the Materials and Methods section (i.e. exactly where I would expect it) the authors present a clear hypothesis that they want to test: whether corrective responses to vibration are larger for narrow than for wide targets. They even specify that they will only consider this interesting if it is an early response. This is again explained in the first paragraph of the Results section. It is therefore clear what should be tested. However, the authors then proceed to test many more things (as many authors do). This raises problems with regards to multiple testing. In the tables alone I count 23 p-values, and not all tests are mentioned in the tables. Thus, if all ‘hypotheses’ that are tested are considered equally important I would expect a correction for multiple testing. Only considering these tests (there are more in the paper), using a Bonferroni correction as suggested on page 28 would mean that only p-values smaller than 0.0022 (0.05/23) can be considered significant. If so, the target size cannot be claimed to influence the response to triceps vibration. I don’t propose to do this correction, because the hypothesis was clear. I just want to indicate why I think that presenting all these tests is a bad idea, irrespective of the space. 

3. Why do the authors provide generalized eta squared as a measure for effect size? They never do anything with these values and they have actual effect sizes in N. 

4. I do not understand the post-hoc tests mentioned on page 28. When there are only two levels there is never a need to do post-hoc tests. 

5. On page 31, when mentioning that the forces are more pronounced for reaches to narrow targets, should the authors not be referring to panels a and d rather than to the left column? 

6. In the last sentence of the abstract: … corrections IS at about 100 ms, … 

------------------------

_____________________________________________________________________________________

Authors’ Response	05/11/18

_____________________________________________________________________________________

# Authors' response to 2^nd^ review comments after 1^st^ revision

05–Nov–2018

We thank the editor and reviewer for the positive evaluation of our manuscript and their helpful comments and recommendations. Below we address all raised points, and underlined the corresponding changes to the text in the revised manuscript. Comments by the reviewer are in *italic*.

## Reviewer: 2

*Comments to the Author
I think this is a good revision. I have a few suggestions but none of them are very important.

1. I would change the title again. It now sounds as if participants are reaching to the muscle vibration. Maybe remove the word ‘reach’. Just “Task-dependent responses to muscle vibration” does not change the intention, I think.*

Reply: We agree with the potential misunderstanding but would like to keep the explicit reference to reaching movements. Hence, we changed the title to “Task-dependent responses to muscle vibration during reaching”. *2. I would remove all the tables. Except for the fact that all the important statistics are also presented in the text (it is up to the editor to decide whether such duplication is acceptable) I think having all these tables emphasizes the sloppy way in which statistical hypothesis testing is currently used in our field (in this and many other studies). On page 19, just before the Materials and Methods section (i.e. exactly where I would expect it) the authors present a clear hypothesis that they want to test: whether corrective responses to vibration are larger for narrow than for wide targets. They even specify that they will only consider this interesting if it is an early response. This is again explained in the first paragraph of the Results section. It is therefore clear what should be tested. However, the authors then proceed to test many more things (as many authors do). This raises problems with regards to multiple testing. In the tables alone I count 23 p-values, and not all tests are mentioned in the tables. Thus, if all ‘hypotheses’ that are tested are considered equally important I would expect a correction for multiple testing. Only considering these tests (there are more in the paper), using a Bonferroni correction as suggested on page 28 would mean that only p-values smaller than 0.0022 (0.05/23) can be considered significant. If so, the target size cannot be claimed to influence the response to triceps vibration. I don’t propose to do this correction, because the hypothesis was clear. I just want to indicate why I think that presenting all these tests is a bad idea, irrespective of the space.*

Reply: We removed all tables and ensured that all information is provided in the text instead (except for effect sizes, see below). *3. Why do the authors provide generalized eta squared as a measure for effect size? They never do anything with these values and they have actual effect sizes in N.*

Reply: We removed the generalized eta squared values from the manuscript. *4. I do not understand the post-hoc tests mentioned on page 28. When there are only two levels there is never a need to do post-hoc tests.*

Reply: We removed all (wording about) post-hoc tests from the main text, and instead only describe the direction of the cell means of (significant) 2-level interactions. *5. On page 31, when mentioning that the forces are more pronounced for reaches to narrow targets, should the authors not be referring to panels a and d rather than to the left column?*

Reply: Indeed, we corrected the text. *6. In the last sentence of the abstract: … corrections IS at about 100 ms, …*

Reply: Corrected in the text.
